# A Titratable Cell Lysis-on-Demand System for Droplet-Compartmentalized Ultrahigh-Throughput Screening in Functional Metagenomics and Directed Evolution

**DOI:** 10.1021/acssynbio.1c00084

**Published:** 2021-07-14

**Authors:** Che Fai Alex Wong, Liisa van Vliet, Swapnil Vilas Bhujbal, Chengzhi Guo, Marit Sletmoen, Bjørn Torger Stokke, Florian Hollfelder, Rahmi Lale

**Affiliations:** † Department of Biotechnology, Faculty of Natural Sciences, Norwegian University of Science and Technology, Trondheim, N-7491, Norway; ‡ Department of Biochemistry, University of Cambridge, 80 Tennis Court Road, Cambridge, CB2 1GA, United Kingdom; ¶ Department of Physics, Faculty of Natural Sciences, Norwegian University of Science and Technology, Trondheim, N-7491, Norway

**Keywords:** single-cell lysis, single-cell screening, droplet screening, microfluidics, functional metagenomics, synthetic biology

## Abstract

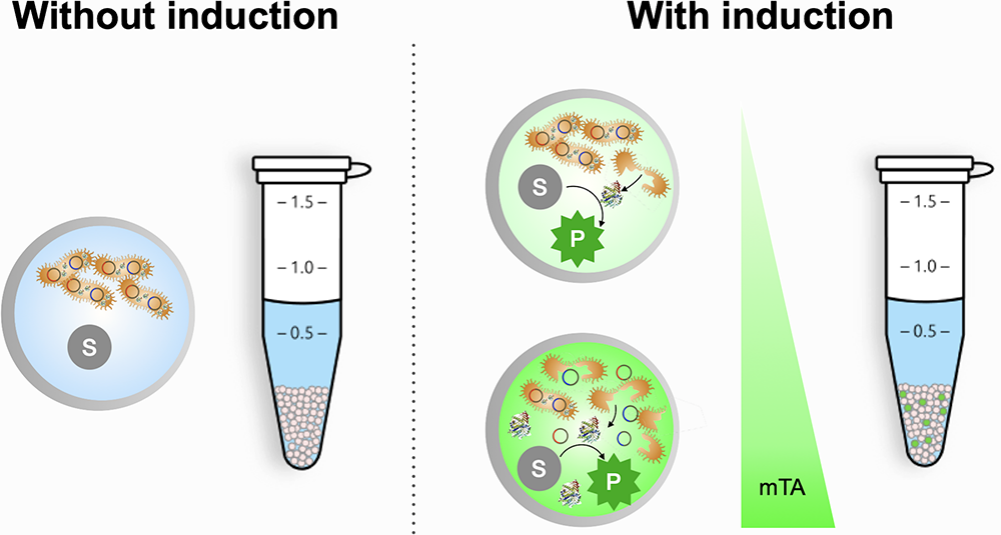

Water-in-oil emulsion droplets are an attractive format for ultrahigh-throughput screening in functional metagenomics and directed evolution applications that allow libraries with more than 10^7^ members to be characterized in a day. Single library members are compartmentalized in droplets that are generated in microfluidic devices and tested for the presence of target biocatalysts. The target proteins can be produced intracellularly, for example, in bacterial hosts in-droplet cell lysis is therefore necessary to allow the enzymes to encounter the substrate to initiate an activity assay. Here, we present a titratable lysis-on-demand (LoD) system enabling the control of the cell lysis rate in *Escherichia coli*. We demonstrate that the rate of cell lysis can be controlled by adjusting the externally added inducer concentration. This LoD system is evaluated both at the population level (by optical density measurements) and at the single-cell level (on single-cell arrays and in alginate microbeads). Additionally, we validate the LoD system by droplet screening of a phosphotriesterase expressed from *E. coli*, with cell lysis triggered by inducer concentrations in the μM range. The LoD system yields sufficient release of the intracellularly produced enzymes to bring about a detectable quantity of product (measured by fluorescence in flow cytometry of double emulsions), while leaving viable cells for the downstream recovery of the genetic material.

Enzymes are biocatalysts that work under varying environmental conditions ranging from mild to extreme. Biotechnological processes can take advantage of such a broad capacity of enzymatic catalysis to meet the goals of carbon neutrality, low energy requirements, less toxic byproducts, and cost competitiveness.^1^ However, currently only a fraction of the processes could conceivably benefit from the large spectrum of biocatalysts, since many enzymes in Nature’s repertoire have not been discovered and/or utilized. Clearly, the potential of enzymes serving as green alternative catalysts has to be realized to achieve and satisfy the ambitions of a sustainable future economy.

Harvesting the metagenome enables us to sample Nature’s unique repertoire of functional molecules. Modern metagenomic studies are accelerated by DNA sequencing and high-throughput screening that allow us to browse and discover the vast microbial diversity from a wide range of environments. Within the field of metagenomics, two main approaches exist: (i) *in silico* approaches, to explore the DNA and/or protein sequences available in private/public databases;^2^ and (ii) functional metagenomics,^3^ the experimental approach to screen for the presence of an enzymatic reaction. In the latter, environmental DNA (eDNA) from microbes^3^ is collected and screened, based on the hypothesis that the environment provides a vast array of coded, useful functions that can be mined. While the diversity of eDNA represents a largely hidden resource for the exploration of this functional repertoire, the functional screening efforts introduce a “needle in a haystack” challenge which results in a typical low hit rate, with an estimate of around one hit per 10^4^–10^5^ variants.^4^

The success of functional metagenomics depends on the quality of the functional assays carried out to identify novel enzymes. Previously, expensive and time- and resource-demanding microtiter plate-based assays (limited to the throughput of ∼10^4^ per day, even with liquid handling robots) were the methods of choice; however, nowadays miniaturized droplet-based microfluidic methods are emerging.^5−7^ Here the screening scale is in the picoliter droplet-range with single-cell encapsulation—ensured via Poisson distribution—to maintain a genotype-to-phenotype linkage.^5,8,9^ In functional metagenomics, microbes serve as living cell factories to transform the genetic information encoded by eDNA into functional enzymes for screening. The model organism *Escherichia coli* is the typical workhorse of choice due to the ease of handling and the availability of a wide range of genetic tools. Using microfluidics as a platform for functional screening, up to 10^8^ reactions can be assayed per day, and sorted at kHz frequencies followed by the recovery of the droplets containing the hits.

The recombinant proteins that are to be screened are mostly produced intracellularly. Therefore, breakdown of the cell barrier is necessary to release the intracellular content to encounter the extracellularly administered substrate. Traditional methods, such as mechanical,^10^ electrochemical,^11^ and thermal^12^ cell lysis are well established for the large-scale extraction of bioproducts. While the above-mentioned crude approaches lead to efficient cell lysis (i.e., via the shearing force, heat, or chemical reagents used), they also destabilize the droplet compartment and possibly compromise product integrity. These methods can be challenging to adapt into microfluidic settings. Instead, several methods were developed to carry out cell lysis in cell-encapsulating droplets, such as chemical,^5,13^ electrical,^14^ thermal,^15^ or traveling surface acoustic wave^16^ intervention. Chemically induced cell lysis is commonly used due to its convenience, but fine-tuning the amounts of reagents needed can be hard. Furthermore, the detergents contained in commercial cell lysis mixtures lead to the destabilization of emulsion droplets. This destabilization can be mitigated by increasing the concentration of stabilizing surfactant; however, higher surfactant concentrations, in turn, lead to higher substrate leakage.^17^

Owing to the presence of genotype–phenotype linkage of the droplet compartment, the “hits” from a functional screening can be directly identified by PCR and subsequent DNA sequencing, as demonstrated in earlier studies.^5,13^ However, the diversity of the resulting “hits” is likely to be restricted by innate PCR-bias. Furthermore, due to inefficient recovery of DNA after screening, this direct approach works best with high copy-number plasmids. The use of low copy-number plasmid leads to low recovery and also decreases the effective throughput due to the loss of clones that are not recovered by PCR. Direct fosmid recovery by PCR is difficult to achieve in practice. To address this potential loss in recovery, it is preferable to obtain viable bacterial host cells for subsequent culturing, from which the hit’s DNA construct can be obtained. Furthermore, the recovered culture can be used immediately for secondary screening, as reported in recent studies.^7,18^ Ideally, a lysis system for microdroplets should provide the user with a tunable control to meet the demand from various assays and, at the same time, enable recovery of survivor cells that can be subsequently isolated for the downstream processes.

In this study, we report the development of a genetic system for controlled lysis of *E. coli* cells in microfluidic droplets, triggered by the addition of a small molecule activator. Such a lysis-on-demand (LoD) system makes intracellular content accessible within the droplets, so that an activity assay can be carried out without jeopardizing the integrity of the droplet compartment. At the same time, the titratable nature of an LoD system enables control over the extent of lysis: leaving intact cells behind, which can then be recovered after droplet sorting to obtain the genetic material.

The LoD system is based on the enterobacteria phage T4 holin–endolysin system.^19^ A wide range of holin–endolysin systems has been characterized and engineered previously, such as the model phage λ^20^ and phage ϕX174^21^ accompanied by different inducible regulatory systems providing a variety of signals for user choice, such as chemical,^22^ UV,^23^ and heat^24^ (see Gao et al.^25^ for a list of different designs). In this study, we used the BioBrick part BBa_K112808 from the Registry of Standard Biological Parts.^26^ The lysis cassette is composed of three coding sequences for the proteins Holin, Endolysin and Antiholin (Figure 1). The first protein, Holin (monomer), accumulates and oligomerizes in the cytoplasmic (inner) membrane which later forms pores through the membrane and allows the second protein, Endolysin, to access and digest the peptidoglycan cell wall layer (periplasm). Because of the weakening of the cell wall, bacterial cell lysis occurs as a consequence of osmotic pressure disrupting the outer membrane integrity. In the LoD system, the expression of *holin* and *endolysin* is controlled by the AraC-XylS transcription factor family member XylS/*P*_*m*_ system. The expression from the *P*_*m*_ promoter can be tightly regulated as a function of the inducer molecule 3-methylbenzoic acid (*meta*-toluic acid, mTA).^27^ The third protein, Antiholin, dimerizes with Holin thus inhibiting the hole-formation in the inner membrane which in turn prevents cell lysis as the access of Endolysin to the periplasm would be blocked. Antiholin is constitutively expressed in order to prevent cell lysis in the uninduced state from any possible leaky expression.

**Figure 1:**
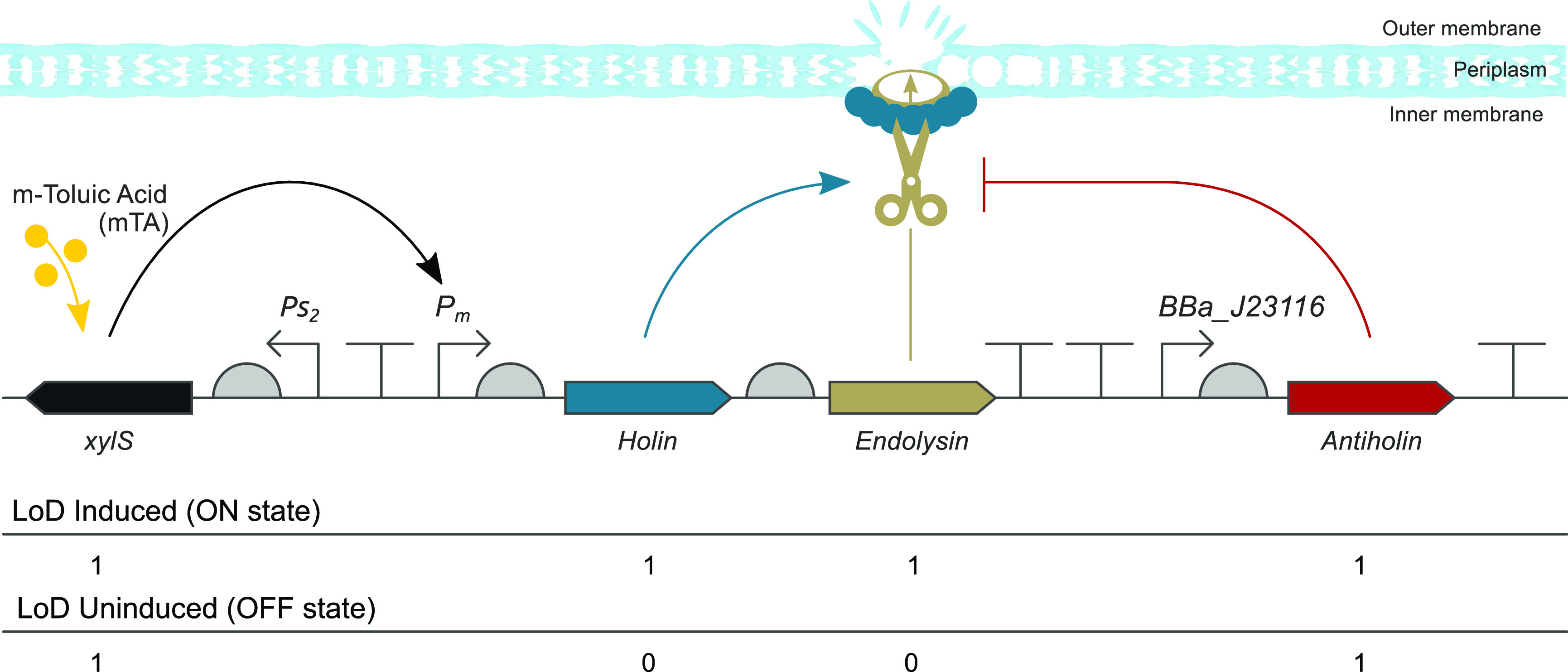
Schematic representation of the lysis cassette, cell lysis process, and truth table. The constitutive promoters *Ps*_2_ and *BBa*_*J23116* control the expression of the transcription factor XylS and Antiholin, respectively. The inducible *P*_*m*_ promoter controls the expression of both *holin* and *endolysin*. The inducer, *meta*-toluic acid (mTA), leads to a conformational change in the transcription factors, XylS, and consequently activation of *P*_*m*_. All four coding sequences are preceded with unique 5′ untranslated regions. The lysis cassette harbors four transcription terminators: one in between the *Ps*_2_ and *P*_*m*_ promoters, two terminators downstream of the *endolysin*, and the fourth downstream of the *antiholin* coding sequences. The truth table is presented below the lysis cassette.

We assess the induction of cell lysis by three methods: first, by spectrophotometric measurement of the total cell density at the population level; second, by following immobilized single cells on a microcontact printed (μCP) pattern (single-cell arrays) to determine the number of live cells upon the induction of cell lysis; and third, by following encapsulated single cells in alginate microbeads (∼50 μm^3^) to demonstrate the cell lysis in droplets. In addition, we validate the use of the LoD system in droplet microfluidics with an assay for a model target enzyme, phosphotriesterase (PTE), by demonstrating titratable intracellular enzyme release upon induction.

## Results

### Construction of a Lysis-on-Demand System

For the development of a LoD system, we used the enterobacteria phage T4 lysis device from the Registry of Standard Biological Parts, BBa_K112808. The DNA sequence of the lysis cassette was synthesized (GenScript Biotech Corporation) and cloned into the plasmid pHH100-mCherry,^28^ that harbors the XylS/*P*_*m*_ system (Figure 1). The coding sequence of the wild type replication protein, TrfA, was replaced by the variant *trfA*-cop271 to increase the copy number of the plasmid from ∼5 to ∼20 copies per chromosome/cell.^29^

### Assessment of Cell Lysis

#### Optical Density and Colony Forming Unit Measurements

**Figure 2:**
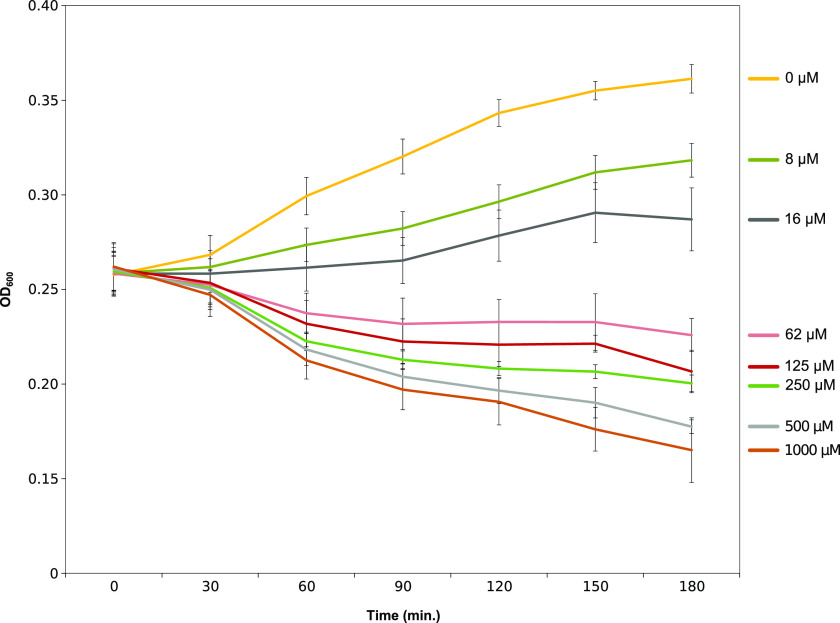
Changes in the optical density (OD_600_) as a function of a range of inducer concentrations tested (8–1000 μM mTA) to trigger the LoD system. The OD_600_ measurements were performed with *E. coli* DH10B-GFP cells harboring the LoD system. The OD_600_ changes were followed, as a proxy for cell count, for 180 min. Error bars represent the 95% confidence interval from triplicates.

*E. coli* DH10B cells, constitutively expressing a chromosomally located green fluorescent protein (GFP) and harboring the LoD system (*E. coli*-GFP-LoD), were grown until they had reached the log phase and were induced with a range of inducer concentrations (8–1000 μM mTA) to trigger the LoD system. The OD_600_ changes were followed, as a proxy for cell count, for 180 min (Figure 2). A decrease in OD_600_ was observed and the rate of decrease corresponded to the level of inducer concentration added. The percentages of OD_600_ reduction at the time point 180 min were 12%, 19%, 24%, 31%, and 35% for the inducer concentrations 62, 125, 250, 500, and 1000 μM mTA, respectively. The OD_600_ for the inducer concentrations 8 and 16 μM mTA was increasing over time; however, both the rate of increase as well as the final OD_600_ at 180 min were lower than that of the uninduced control sample, suggesting cell lysis despite the cell growth. The concentration-dependent reduction in OD_600_ indicated that the cell lysis is titrable and the amount of cell lysis can be controlled by adjusting the inducer concentration.

The colony forming units (CFUs) provide a direct assessment of cell viability. The same range of inducer concentrations tested for OD_600_ measurements (8–1000 μM mTA) were used in assessing the CFU change over time upon induction of the LoD system. *E. coli*-GFP-LoD cells were sampled with 60 min interval for 180 min and were plated on agar plates (Table 1). After 180 min of induction, a decrease in CFU counts was observed in all but the samples induced with 0, 8, and 16 μM mTA, and the amount of decrease roughly corresponded to the concentration of inducer added. The percentage of survivors, as determined by dividing the initial CFU counts over the final CFU, were 27.8%, 3.1%, 1.2%, 1.5%, and 0.8% for the inducer concentrations 62, 125, 250, 500, and 1000 μM mTA, respectively. Similar to the OD experiment (Figure 2), an increase of CFU was observed in the two lowest inducer concentrations, 8 and 16 μM mTA.

**Table 1:** Colony Forming Unit (CFU) Counts (per mL) Change over Time of *E. coli*-GFP-LoD Cells Induced with Various Inducer Concentrations (mTA). The CFU Counts Are Shown as Mean and ± SD from Triplicate Experiments

inducer concn (μM)	0 min	60 min	120 min	180 min
0		(5.0 ± 3.6) 10^6^	(2.4 ± 0.4) 10^7^	(4.8 ± 1.9) 10^7^
8		(3.1 ± 2.3) 10^6^	(1.3 ± 1.0) 10^7^	(4.3 ± 0.4) 10^7^
16		(1.8 ± 1.9) 10^6^	(1.2 ± 0.8) 10^7^	(3.1 ± 0.7) 10^7^
62		(1.5 ± 0.5) 10^5^	(2.1 ± 0.2) 10^5^	(1.2 ± 0.8) 10^5^
125	(4.3 ± 1.3) 10^5^	(2.6 ± 0.8) 10^5^	(1.4 ± 0.4) 10^5^	(1.3 ± 0.2) 10^4^
250		(2.3 ± 0.9) 10^5^	(4.3 ± 0.6) 10^4^	(5.3 ± 1.5) 10^3^
500		(2.9 ± 1.3) 10^5^	(4.8 ± 1.0) 10^4^	(6.6 ± 3.0) 10^3^
1000		(2.9 ± 0.9) 10^5^	(2.3 ± 0.7) 10^4^	(3.5 ± 0.5) 10^3^

#### Immobilized Single-Cell Counts

**Figure 3:**
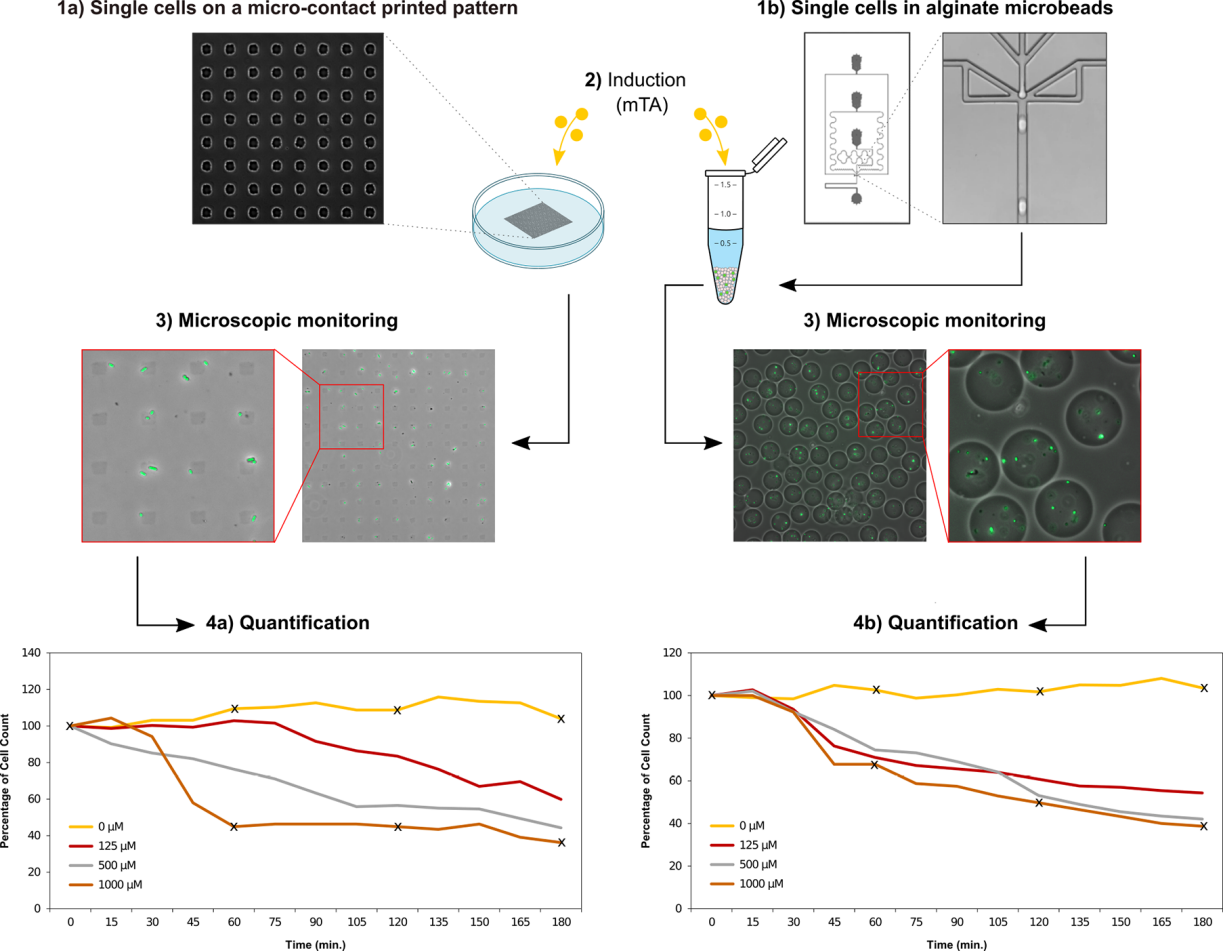
Schematic workflow describing the single-cell measurements. Single *E. coli*-GFP-LoD cells were (1a) immobilized on a μCP PEI-coated pattern (single-cell arrays) and (1b) encapsulated in alginate microbeads. (2) Both systems were induced with a range of inducer concentrations (0, 125, 500, and 1000 μM mTA). (3) Single cells were analyzed based on time-lapse fluorescence microscopy. Image analysis was performed to quantify the rate of the cell lysis based on the GFP intensity measurements from single cells on (4a) single-cell arrays and (4b) encapsulated in alginate microbeads over 180 min under different inducer concentrations. The analyzed microscopic images at the cross-marked time-points (0 and 1000 μM at 60 min intervals) are displayed in Figures S1 and S2.

Log phase *E. coli*-GFP-LoD cells were immobilized on a μCP polyethylenimine (PEI)-coated pattern (single-cell arrays) and characterized by time-lapse fluorescence microscopy (Figure 3(1a); Figure S1). The number of single cells were determined using the captured images at the specified sampling time points (0 to 180 min with 30 min intervals), as detected by the individual GFP signals. The loss of GFP/cell counts indicated a cell death event due to the lysis of the immobilized cells, leading to the release of the intracellular GFP into the background.

The GFP expression values from the immobilized cells at the time point 0 were recorded for each of the three inducer concentrations tested. The relative percentage of the lysed cells (the disappearance of the GFP signal) at different sampling time points were quantified relative to the time point 0. After 180 min, the relative percentage of the dead cells were 40%, 56%, and 64% for the inducer concentrations 125, 500, and 1000 μM mTA, respectively.

#### Encapsulated Cells in Alginate Microbeads

**Figure 4:**
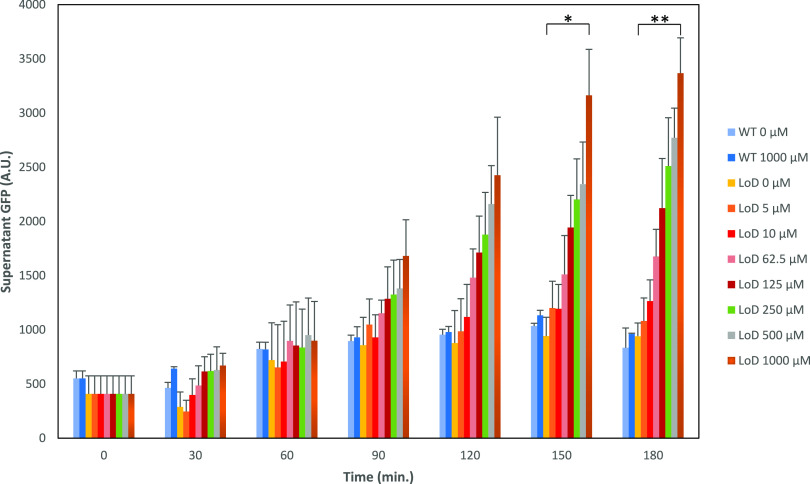
Measured GFP in the supernatant upon induction of the LoD system. Filtrated supernatant from *E. coli*-GFP cells with (LoD) or without the LoD system (WT) were measured every 30 min. Error bars represent the standard deviation from triplicates. The fluorescence is given in arbitrary units (A.U.) A significance analysis was performed for the samples WT 0 μM and LoD 1000 μM at the time points 150 and 180 min. Asterisks indicate the results of *t* tests, *P-value < 0.001; **P-value < 0.0003.

Log phase *E. coli*-GFP-LoD cells were encapsulated in alginate microbeads and characterized by time-lapse fluorescence microscopy (Figure 3(1b)). The cell concentration was adjusted to ensure that each microbead contained more than a single-cell. In the three inducer concentrations tested, the final amount of cell lysis at 180 min was similar to the levels obtained with the single-cell arrays, at around 46%, 58%, and 62% for 125, 250, and 1000 μM mTA, respectively. There were, however, a few differences observed in comparison to the single-cell array results: first, the beginning of lysis in the alginate microbeads was observed at 30 min rather than at 15 min (Figures 3 and 4a,b; Figure S2); second, the difference in the percentage of lysis observed between the different inducer concentrations became less noticeable over the course of 180 min. We speculate that this observed difference is due to the lower growth observed for the cells encapsulated in alginate microbeads as compared to those grown in batch or on single-cell arrays.

#### Assessment of Cell Lysis by Fluorescent Protein Release Assay

To demonstrate the functionality of the LoD system, the release of the intracellularly produced GFP followed upon induction. *E. coli*-GFP-LoD cells were induced with different concentrations of mTA, and GFP intensity measurements were carried out in the filtered growth medium (Figure 4) for 180 min with 30 min intervals. *E. coli*-GFP cells without the LoD system, uninduced and induced (1000 μM mTA), were used as negative controls. An increase of GFP readings in the cell-free medium was already observable after 90 min of induction, but statistically significant differences were only detected later on, first at 150 min (1000 μM vs 0 μM, *P*-value < 0.001), and maintained further until the end (*P*-value < 0.0003), whereas the GFP readings in the negative controls remained relatively stable.

#### Release of Phosphotriesterase into Double-Emulsion Droplets

**Figure 5:**
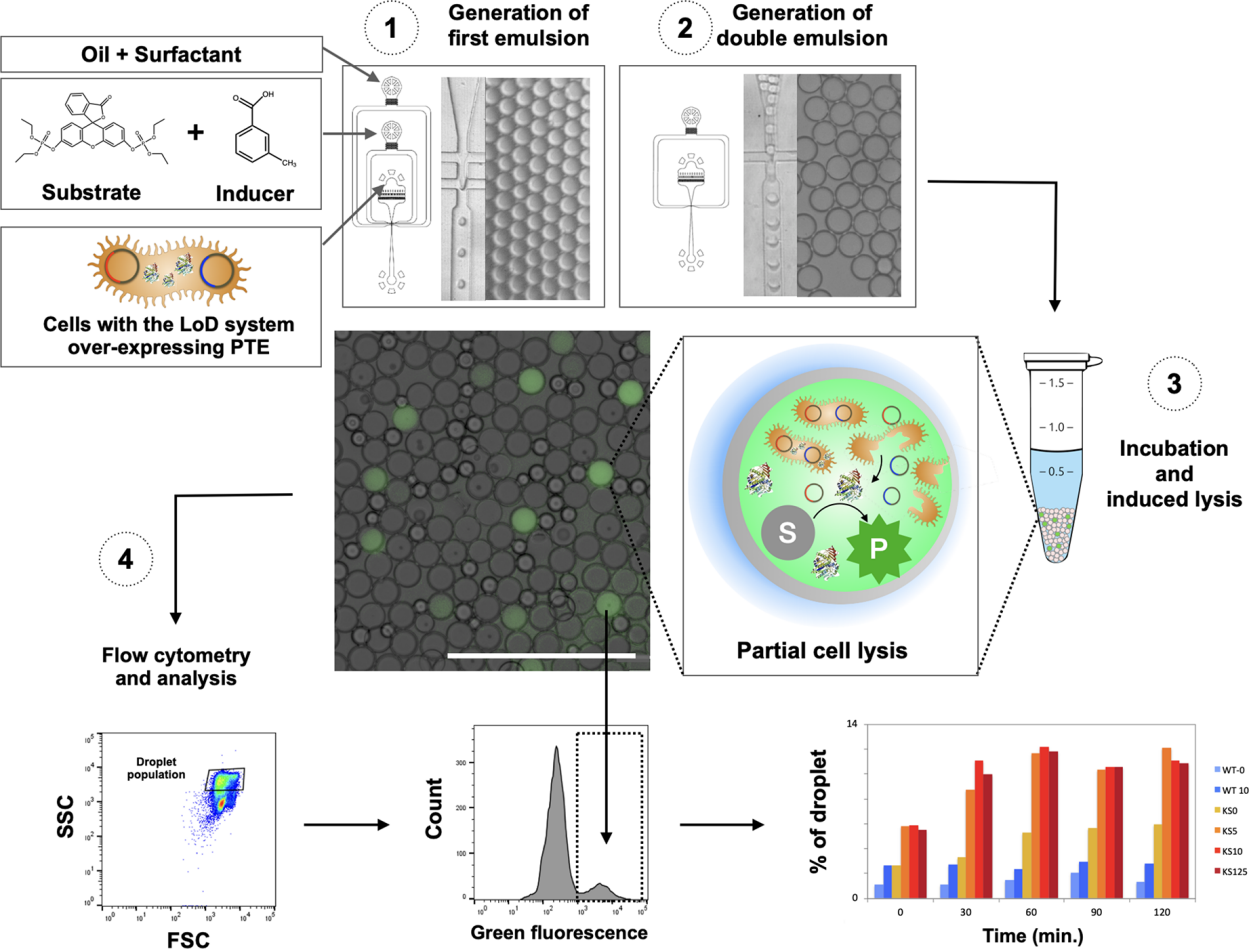
Workflow describing the double-emulsion assay. (1) Fluorogenic substrate (10 μM fluorescein didiethyl phosphotriester [FddEP]), *E. coli*-PTE-WT and *E. coli*-PTE-LoD cells, and the inducer (0, 5, 10, and 125 μM mTA) were encapsulated into a fluorinated oil and surfactant phase (0.5% fluorosurfactant-008 in HFE-7500) to generate 7 pL microfluidic droplets in a flow-focusing device (channel width: 18 μm). (2) The emulsion was reinjected into a second device to generate water–oil–water double emulsion droplets that were (3) incubated (0, 30, 60, 90, and 120 min at 37 °C) to allow for induction of lysis and conversion of the fluorogenic substrate into product as shown in the overlay photograph. (4) For each time point, aliquots of the double emulsion were analyzed by flow cytometry. The population of droplets was gated on the side- and forward-scattering signals (SSC and FSC), as shown in the SSC vs FSC plot, and analyzed to quantify the ratio of green fluorescence signal between the two droplet populations: with and without reaction product. Events with lower SSC signal were excluded as they represent smaller oil droplets produced during the second emulsion generation (visible in the photograph). The percentage of droplets in the high fluorescence population was quantified over time as a function of a variety of conditions *E. coli*-PTE-WT and *E. coli*-PTE-LoD cells at different inducer concentrations) to be explored.

After demonstrating the GFP release upon cell lysis, a workflow for single-cell compartmentalization^30^ and lysis^13^ was designed that allowed monitoring of reaction turnover of a PTE, a representative hydrolase and a target of functional metagenomic screens^5^ (Figure 5). To this end, water-in-oil emulsion droplets were generated in a polydimethylsiloxane (PDMS) device. Droplets were formed by break-off flow using an aqueous phase containing *E. coli* cells constitutively expressing PTE with (*E. coli*-PTE-LoD) and without the LoD system (*E. coli*-PTE-WT), and a second aqueous phase containing both the substrate and inducer, while the fluorous oil carrier phase contained the surfactant. For ease of analysis, the single emulsion droplets were directly re-emulsified in a second device to form water-in-oil-in-water microdroplets (Figure S3), as these double-emulsions are amenable to flow cytometric sorting.^6,7^

**Figure 6:**
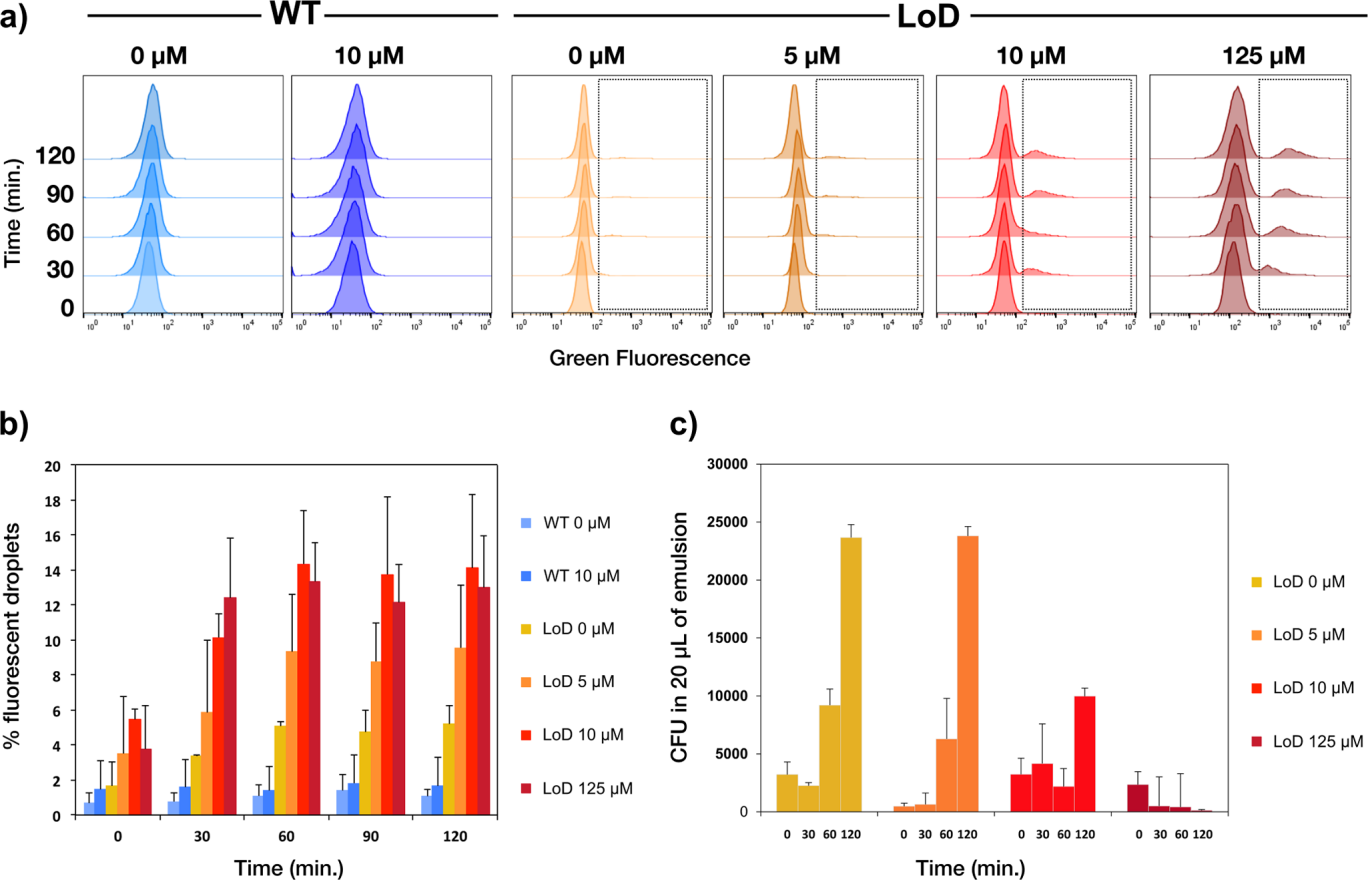
Flow cytometry analysis of the double-emulsion droplets. Droplets containing the *E. coli*-PTE-WT and *E. coli*-PTE-LoD cells with various concentrations of the inducer were incubated at 37 °C and analyzed by flow cytometry. (a) The fluorescence histograms for each condition show a second higher fluorescence population (highlighted in a box) for the *E. coli*-PTE-LoD but not for the *E. coli*-PTE-WT cells. (b) The percentage of droplets above the main droplet population without fluorescence over the time-course of the assay with the thresholds determined at minima between the two populations for each condition. (c) The average cell recovery from *E. coli*-PTE-LoD cells induced with 0, 5, 10, and 125 μM mTA in colony forming units (CFU) from 20 μL of droplets (diluted 100 times) incubated for 120 min. Error bars represent the standard deviation of three dilutions, each in triplicate.

When the double emulsion droplets were analyzed (Figure 6a), a population with high fluorescence could be detected among the droplets that were containing the *E. coli*-PTE-LoD cells, saturating after the completion of the enzymatic turnover (achieved by 60 min of incubation). While the *E. coli*-PTE-LoD cells were induced with a range of inducer concentrations (0, 5, 10, and 125 μM mTA), the control cells (*E. coli*-PTE-WT) were induced with no (0 μM) or 10 μM mTA. The fluorescence levels of the droplet populations were measured over 120 min (Figure S5). The proportion of droplets correlated to the envisaged Poisson distribution (theoretically 22% droplets with single-cell occupancy), consistent with a scenario in which all cells eventually lyse in the presence of the inducer. Droplets containing *E. coli*-PTE-WT cells did not show a significant population of high fluorescence droplets suggesting that lysis occurs only in the presence of the LoD system. *E. coli*-PTE-LoD cells in the uninduced-state show a background of weak, leaky expression that lead to a small amount of product turnover with up to 5% of droplets containing the released product. This increased to 10% of the droplet fraction turning over product in droplets containing 5 μM of mTA. In the presence of 10 μM and 125 μM mTA a plateau of 14 ± 4% fluorescent droplets was observed (within error and unavoidable losses compared to the theoretical fill), suggesting that 10 μM is sufficient to fully lyse the cells (Figure 6a,b). Despite the use of such a low inducer concentration the cell lysis is still titratable (Figure S6) with almost full lysis obtained at 10 μM of mTA. In addition, the coefficient of variation for the fluorescent population (Figure S8) does not vary much across inducer concentrations, suggesting that the LoD system behaves uniformly across the droplets.

#### Recovery of Cells from Droplets

The main objective of functional metagenomic screening is to recover the genetic material associated with the screened phenotype. While the recovery of high-copy number plasmids in droplets is achievable,^31,32^ it is challenging to recover low-copy number plasmids or larger DNA constructs, such as fosmids and cosmids^7^ from the cell lysates in droplets. As a remedy to this problem, growing the recovered cells can ease and enhance the recovery of the genetic material.

Controlled cell lysis of a titratable nature would therefore allow us to tune the amount of the enzyme available in an assay reaction in droplets, while leaving intact cells behind to be recovered after screening instead of relying on the spontaneous lysis of cells.^7^Figure 6c shows that during the 120 min incubation of the droplets, those containing 5 and 10 μM mTA not only resulted in the recovery of *E. coli*-PTE-LoD cells but also showed cell growth with an increase in CFU over time; by contrast, in the droplets containing 125 μM mTA, the number of cells recovered decreases over time, which implies cell death and complete lysis. This correlates with decreasing growth (measured by OD_600_ and cell lysis measurements [Figures 2 and 3]) and with the observation of high fluorescence intensity of the droplets containing the *E. coli*-PTE-LoD cells induced with 125 μM mTA (Figure S7C).

## Discussion

In this study, controlled cell lysis with the LoD system was tested with a wide range of methods and inducer concentrations spanning from the maximal concentration at 1000 μM mTA in microcentrifuge tubes, on single-cell arrays, and in alginate microbeads, to almost two hundred-fold dilutions at 5 μM mTA in double emulsion droplets. Intriguingly, the titratable nature of the LoD system could still be observed at the low inducer concentrations in the 5–10 μM range in the double emulsion droplets. The induction characteristics of the XylS/*Pm* system at such low levels of inducer concentrations have not been reported previously. While the low levels of mTA (e.g., 10 μM) show no apparent effect in the bulk measurements, droplet compartmentalization reveals cell lysis of a fraction of the clones as a single-cell effect that is invisible in the population level measurements (Figure 6). The effects of low levels of inducer concentrations would thus not be possible to capture without the use of microfluidics. The induction of the LoD system is fast, as the rapid PTE reactions can be detected already after 30 min of induction. Using LoD, we can design precisely timed workflows that take account of cell growth rates (to produce sufficient amounts of enzyme), the extent of lysis (to preserve a sufficient number live cells for recovery), set up a favorable ratio of the signal from the enzymatic reaction against the background reactions (uncatalysed and cellular) and trigger enzyme release at once (compared to much slower release of enzyme by spontaneous lysis^7^).

A key objective of the LoD system is the controlled release of the intracellular product, while leaving intact cells that can be recovered and regrown after sorting. In the PTE assay, the enzymatic signals were detected from 30 min onward without reduction in CFU counts at the lowest inducer concentration tested (5 μM mTA). In fact, after 120 min, the cell counts with 5 μM mTA induction had gradually increased to a similar level as the uninduced control. Since it is doubtful that the *E. coli* would be recoverable if the peptidoglycan layer was degraded beyond repair, that is, as a consequence of a bursting of cell due to osmotic pressure, it appears that intracellular leakage of enzyme is occurring without cell death. At such low inducer concentration, it is possible that the weakly expressed Endolysin is insufficient to cause cells to burst, as the peptidoglycan layer is known to be an active dynamic structure. However, additional work is required to clarify the exact cell status. Regardless, *E. coli* cells are reliably recoverable, even when the LoD system was induced with 125 μM mTA, despite lower CFU counts obtained after 120 min compared to the samples induced with 10 μM mTA.

While we demonstrate the functionality of the LoD system in *E. coli*, the most widely used bacterial host for functional screening, the system can also be adapted to other Gram-negative bacterial hosts. Holin–Antiholin systems are known to be functional in several Gram-negative bacteria, such as *Halomonas campaniensis* LS21^33^ and *Pseudomonas putida*,^34^ which would provide alternatives in case heterologous expression in *E. coli* fails to express the target biocatalysts. While the XylS/*Pm* system is known to work in multiple bacterial hosts^35^ the induction requires the presence of a passively diffusing inducer molecule. In future applications, a system that is not dependent on an externally added molecule might be advantageous. For instance, a simplified activation of cell lysis could be envisioned by the use of optogenetics: the activation of cell lysis could be controlled by a light-inducible promoter, not requiring the addition of an inducer, which could potentially simplify the workflow of microfluidic s-based screening yet maintain the benefit of tunability. Early versions of a light-sensitive expression system such as EL222^36^ had major drawbacks, such as low-fold changes between the ON/OFF status; however, a recent study reports an engineered light sensor system, RsLOV,^37^ that can provide highly tunable expression with levels comparable to the widely used T7 expression system.

The use of a LoD system will facilitate future droplet screening efforts compared to the current chemical lysis protocols. It is known that surfactant components in lysis kits destabilize droplets^13^ and they also lead to increased leakage of reaction products from droplets.^17^ Importantly, the exact composition of commercial cell lysis kits are not available in detail, making it difficult to mitigate their effects and safeguard against batch-to-batch variations. Eliminating such complications could enable longer incubation periods (to find enzymes with initially low activity, for example, when a weak promiscuous activity is enhanced^38^ or in metagenomic screening^5^). Chaotropic agents in lysis mixtures are also known to adversely affect downstream assay performance,^39,40^ and lysis regents can cause direct chemical damage to the microorganisms.^39^

For droplet-compartmentalized experiments that start with Poisson distributed single cells and are followed by “monoclonal” cell-growth in droplets^18^, the concentration-dependent modulation of cell lysis will provide a useful level of control. For example, most droplet-based experiments employ high-copy number plasmids, to improve the recovery of the genetic material. Now with the reported LoD system, the rate of cell lysis can be controlled by adjusting the inducer concentration to bring about a suitable proportion of the lysed cells to detect the product and also capture the DNA sequence from each of the selected target clones directly from the cells, without an additional transformation step^5,13,41^ that incurs loss of DNA.

Droplet compartmentalization of single genes followed by *in vitro* expression is an alternative to protein production by cells. A number of studies have successfully used this format for directed evolution in polydisperse droplets^42^ but an equivalent complete directed evolution experiment in monodisperse droplets has only been reported recently.^43^ Here the incompatibility of the DNA amplification, *in vitro* expression and assay condition, necessitates the establishment of a relatively complex workflow with multiple picoinjections of reagents. By using the LoD system described in this study, nonspecialist laboratories without extensive microfluidic expertise can implement a simple workflow with reduced complexity, using single compartmentalization in a standard flow focusing device followed by a further emulsification prior to flow cytometric sorting.^6,7^ A dilution of *E. coli*-PTE-LoD in *E. coli*-PTE-WT (1:1000) was screened (as previously described^5,13^) to mimic a screening campaign (Figures S9 and S10), and confirmed that the LoD system works even in the context of detecting rare events.

A cell-based alternative to lysis protocols are compartmentalized display systems as shown by directed evolution studies using yeast^44^ or bacterial^45^ display. However, not every enzyme is amenable to be displayed on the cell surface and the number of the cell-displayed molecules may be considerably lower than the amount of enzymes that can be produced intracellularly. For example, *E. coli* has been shown to express well above >10^5^ enzyme molecules,^46^ which can be increased by droplet cell growth,^18^ while yeast display may be limited to 10^4^ molecules per cell.

In conclusion, we establish a LoD system suitable for microfluidic ultrahigh-throughput metagenomic screening. The combined use of an established lysis cassette and a sensitive expression system offers a versatile control over cell lysis within droplets, still leaving behind recoverable cells. Both factors of tight control and recovery are currently lacking in the common alternative lysis methods. By adjusting the inducer concentration, the user can control both the amount and speed of cell lysis according to their different screening condition needs. The simplistic design and the use of an inexpensive passively diffusing inducer should allow this system to be readily adaptable into existing microdroplet-based screening in functional metagenomics, and also in directed protein evolution applications.

## Experimental Section

### Bacterial Strains and Growth Conditions

Unless otherwise stated all *E. coli* strains were grown in Lysogeny Broth (LB) medium (10 g L^–1^ tryptone; 5 g L^–1^ yeast extract; 5 g L^–1^ NaCl) at 37 °C with shaking for overnight. The overnight culture was diluted by a hundred-fold in fresh media, and returned to incubator until the log phase was reached. When needed, antibiotics kanamycin and carbenicillin were added to a final concentration of 50 μg mL^–1^. Chemically competent *E. coli* strain DH5α was used for routine molecular cloning. To provide GFP for microscopic monitoring, *E. coli* strain DH10B genotype arsB::cat sfGFP (DH10B-GFP) which constitutively expresses the superfolder (sf)GFP from its chromosome was used (a gift from Dr. Joseph White). In brief, a chloramphenicol resistance sfGFP cassette was recombined into an arsenic resistance gene, *arsB*, using lambda red recombination system. Lastly, it is important to mention that freshly transformed cells should be used, since long-term storage at −80 °C can impair the titratability of the LoD system.

The phosphotriesterase (PTE) gene was obtained in its pET expression vector^47,48^ prior to transformation with LoD plasmid into *E. coli* BL21 (NEB) cells. Positive LoD transformants harboring both expression constructs were selected on LB agar plates supplemented with the LoD antibiotic cocktail (50 μg mL^–1^ carbenicillin and 50 μg mL^–1^ kanamycin). Selected LoD transformants were grown in LB liquid medium with antibiotics (37 °C, shaking). PTE expression was induced with 50 μg mL^–1^ of ZnCl_2_ at OD_600_ of 0.6. After 16 h of induction, cells were harvested and washed twice with LB by centrifugation before dilution to OD_600_ 0.415 in 20% (v/v) Percoll (Sigma-Aldrich) to prevent cell aggregation. Control cells harboring only pET-PTE were selected and prepared by identical procedures other than using only 100 μg mL^–1^ of carbenicillin for antibiotic selection.

### Plasmid Construction

The enterobacterial phage T4 lysis device from Registry of Standard Biological Parts, BBa_K112808, was synthesized (GeneScript) and cloned into the plasmid pHH100-mCherry^28^ using the restriction enzymes NdeI and *Bam*HI. To increase the plasmid copy numbers, the coding sequence of the replication protein, TrfA, was substituted with the *trfA*-cop271 variant.^29^ This cloning was done using restriction enzymes *Bam*HI and PvUII, creating the plasmid pHH-eT4LoD-cop271 (LoD).

### Bacterial Lysis Measurements

To determine the effect of the LoD system, the amount of bacterial lysis under different inducer concentrations was measured in four ways: (1) The changes of optical density at 600 nm (OD_600_), (2) the count of individual immobilized bacterial cells on single-cell arrays, (3) the count of encapsulated bacteria in alginate microbeads, and (4) the released amount of intracellularly produced GFP into the supernatant. All the above experiments were carried out with cultures in the log phase.

To measure the changes of OD_600_, *E. coli* cells DH10B-GFP with plasmid pHH-eT4LoD-cop271 was inoculated in 5 mL of LB overnight. The culture was diluted a hundred-fold in 20 mL of LB with kanamycin in an Erlenmeyer flask and grown for 3 h to reach the log phase. From the log phase culture, 99 μL of cells were aliquoted to a flat-bottom microtiter plate (Thermo Fisher Scientific) and 1 μL of corresponding inducer was added. In total, eight different concentrations of the inducer were tested (0, 8, 16, 62, 125, 250, 500, and 1000 μM mTA). A blank well with 100 μL of LB was included for accounting the background, and all the inducer concentrations were tested in biological triplicates. The OD_600_ of the cultures was measured without the plate lid in a Tecan infinite M2000 Pro (Tecan Life Sciences) at 37 °C with the following setting: linear shaking (15 s, 3 mm amplitude), 5 s waiting time, and absorbance reading at OD_600_ at every 30 min for 180 min.

To measure the amount of intracellular GFP release into the supernatant, log phase *E. coli* culture was aliquoted for the different inducer concentrations. To induce, 100 μL of the inducer stocks (100×) was added to each of the 10 mL aliquoted cultures. After aliquoting, the cultures were returned to 37 °C incubator with shaking. At every 30 min after induction, 1 mL of cultures was collected from each sample and was centrifuged at maximum speed for 1 min in a benchtop centrifuge. To ensure the measured GFP signal is due to the released proteins rather than from residual bacteria, the supernatant was collected and syringe filtered via 0.2 μm pore size membrane (Merck). A 100 μL aliquot of the filtrate was transferred to a flat-bottom Black/Clear 96 wells microtiter plate (Thermo Fisher Scientific), and the amount of GFP intensity was measured with the plate reader Tecan infinite M2000 Pro (Tecan Life Sciences) using the following setting: 488 nm excitation, 530 nm emission, and a manual gain of 90. A control with *E. coli* cells not harboring the LoD system was included and the experiment was carried out in biological triplicate. Student’s *t* test was performed using the GFP values to detect a significant difference between induced and uninduced samples.

### Design and Fabrication of Bacterial Single-Cell Array Stamps and Microfluidic Devices

The bacterial single-cell array stamps for 7 μm square features separated with 14 μm, and microfluidics chip with three inlet and one outlet with a junction diameter of 30 μm were designed in a layout editor software (CleWin, version 4.3.5.0). The microfluidic devices used for the droplet assays were designed by CAD (AutoCAD, Autodesk and DraftSight, Dassault Systemes): A microfluidic device design with a flow focusing junction of 24 μm (height and width) was used with three inlets for droplet generation and a flow focusing junction of 18 μm with two inlets for the double emulsion, as shown in Figure S4. The designs for these devices are freely available to download as CAD-compatible or PNG files from the DropBase Repository of droplet microfluidic device design. The stamp design was replicated to form a single-cell array of nine times repeating 20 × 20 spots. The stamps and microfluidic devices were fabricated by standard maskless soft lithography using a 4 in. silicon wafer. Briefly, the wafer was first washed with acetone followed by isopropyl alcohol (IPA) and finally dried using nitrogen gas. The dried wafer was ozone treated (Novascan) for 3 min, followed by a dehydration bake at 180 °C for 20 min. The dehydrated wafers were spin coated for 33 s at 3000 rpm using negative photoresists mr-DWL5 for a bacterial array and mr-DWL40 for a microfluidics device (Microresist Technology GmbH, Germany). Soft baking of the resist was done by gradually increasing the temperature of the hot plate from 50 to 90 °C. The mr-DWL5 resist was baked for 2 min while the mr-DWL40 was baked for 10 min. The soft-baked resists were gradually cooled on the hot plate by decreasing the temperature to 50 °C, followed by relaxation time of 10 min for mr-DWL5 and 1 h for mr-DWL40 at room temperature. The resist was exposed to UV light (UV 405 nm) using a maskless aligner (Maskless Aligner 150, Heidelberg Instruments, Germany) to directly transfer the design on the resists. The exposure energy was set to 400 mJ cm^–2^ for mr-DWL5 and 500 mJ cm^–2^ for mr-DWL40. The postexposure bake was carried out using an approach that was similar to the one used for the soft bake. Relaxation time after the postexposure bake was set to 1 h for mr-DWL5 and 2 h for mr-DWL40 at room temperature. The resist was developed using mr-Dev 600 (Micro Resist Technology GmbH, Germany). The developer was left 2 min for mr-DWL5 and 6 min for mr-DWL40 using constant stirring. The developed wafers were thoroughly washed in IPA and dried using nitrogen gas. The master molds for the PTE assay and cell recovery from droplets were produced via the soft-lithography method^49^ using high-resolution acetate masks (Microlithography Services Ltd.) and SU-8-2025 photoresist (A-Gas Electronic Materials Ltd.) as previously described.^6,7,50^ The wafers with the developed master molds were then treated with fluorosilane (1*H*,1*H*,2*H*,2*H*-perfluorooctyl(trichlorosilane)) for 1 h in a vacuum chamber to avoid adhesion of PDMS (Dow Corning). PDMS with 10 wt % initiator (Sylgard 184 kit, Dow Corning) was thoroughly mixed for 5 min and degassed for 20 min. The degassed PDMS was casted onto the silanized wafers in a Petri dish and baked (3 to 12 h, 65 °C). The PDMS was subsequently peeled off and used for μCP and microfluidic devices. The PDMS microfluidic devices were punched to create 1 mm diameter holes to enable connection of plastic tubes at the inlets and outlets. The feature side of the PDMS microfluidics devices were plasma treated using a plasma cleaner (20 s, Femto, Diener Electronics) and bonded to glass slides. The bonded PDMS microfluidic devices were baked for 24 h at room temperature, and prior to the alginate cell encapsulation, they were treated with 1% (v/v) of fluorosilane in hydrofluoroether (HFE7500, 3M, Novachem, 5 min). The oil was removed by blow drying with nitrogen gas. The devices for the droplet assay were treated directly after plasma bonding. The triple–inlet chips were flushed with fluorinatedoil (1% v/v in HFE-7500, 3M, Fluorochem) to confer a hydrophobic coating to the channels and baked on a hot plate (80 °C, 20 min). A hydrophilic coating was added to the two-inlet devices used to generate the second or water-in-oil-in-water emulsions, as previously reported.^6,51^ Briefly, immediately after plasma bonding, the devices were incubated on a hot plate (100 °C, 10 min). The devices were then flushed in sequence with poly(diallyldimethylammonium chloride) in 0.5 M NaCl (PDADMAC, Sigma, 2 mg mL^–1^, 10 min), 0.1 M NaCl to rinse, poly(styrenesulfonate) in 0.5 M NaCl (PSS, Sigma, 2 mg mL^–1^, 10 min) and rinsed with DI water. The devices were stored in a sealed and water-saturated box until use.

### On Chip Alginate Cell Encapsulation via Competitive Ligand Exchange Cross-Linking

Alginate gel microbeads were produced on chip using competitive ligand exchange cross-linking (CLEX) method implemented in microfluidic chips as previously described.^52,53^ Briefly, two dispersed phases were used: (1) 0.6% (wt) alginate (Pronova UP LVM, FMC Biopolymer AS, Norway) containing 84 mM CaEDTA and 40 mM MOPS at pH 6.7 and (2) 0.6%(wt) alginate containing 84 mM ZnEDDA and 40 mM MOPS at pH 6.7 with cells. The two dispersed phases met in a coflow region in the microfluidic channels prior to droplet formation in the flow-focusing region. The flow rates were set to 650 μL h^–1^ for the continuous phase (Pico-Surf, Sphere Fluidics) and 50 μL h^–1^ for both dispersed phases by controlled injection using BD plastic syringes mounted on syringe pumps (Harvard Apparatus, PHD ULTRA). We injected cells in the first dispersed phase that was continuously stirred using a small magnet in the syringe to avoid cell sedimentation. The gel beads were recovered from the collected alginate bead emulsions following destabilization of the emulsion (Pico-Break1, Sphere Fluidics) and then transferred to cell culture media until further use for μCP.

### Immobilization of *E. coli* Cells on Single-Cell Arrays and in Alginate Microbeads

To obtain an array of single cells, cytophilic chemical PEI (Mw 750.000 by LS, 50 wt % in H_2_O, Sigma-Aldrich) was deposited using μCP onto glass slides precoated with cytophobic chemicals, as previously described.^54^ Micrometer-sized patterned spots were introduced through μCP based deposition of PEI on glass surfaces passivated through coating with the cytophobic chemical PEG. The surface modifications were carried out as follows: Wilco dishes were first assembled according to the specification by the manufacturer (WillCo Wells B.V.). The glass slides were rinsed with 70% ethanol followed by Milli-Q water and blow-dried with nitrogen gas. The glass slides were covered with a solution containing PLL (20 kDa)-*g*-PEG (2 kDa) 0.1 mg mL^–1^ dissolved in 10 mM HEPES, pH 7.4, for 60 min. The slides were subsequently rinsed in Milli-Q water and dried with nitrogen gas. PDMS stamps and the procedure described in the following were used to introduce patterns of PEI. PDMS stamps were incubated with aqueous 1 wt % PEI for 60 min at room temperature. The stamps were then blow dried using a stream of nitrogen gas and placed pattern-side down on the PEGylated glass slides for 60 min with a 100 g weight on top. The PDMS stamps were then carefully removed from the glass surface, leaving the PEI surface spots arranged in an array as dictated by the design structured in the PDMS stamp. The arrayed surface was immediately covered with 200 μL of dispersed log phase (OD_600_ < 0.3) *E. coli* cells or *E. coli* cells encapsulated in alginate microbeads for 10 and 20 min, respectively. Unattached bacteria or alginate microbeads were removed from the bacterial array by gentle flushing with LB medium. The arrayed surface was immediately covered with culture medium and imaged using fluorescence microscopy at room temperature.

### Fluorescence Microscopy and Image Processing

An inverted microscope (Axio Observer.Z1 from Zeiss, 2.3.64.0) with 20× air objective (NA 0.8) was used for image acquisition. A GFP filter was used when inspecting the viability of the immobilized single cells and cells encapsulated in alginate microbeads. Both immobilized single cells and cells encapsulated in alginate microbeads were imaged in time series for 180 min with an interval of 15 min. In addition to time series, cells were also imaged in the *Z* axis. The *Z* stack of the entire cells and alginate encapsulated cells were obtained using 1 and 5 μm intervals, respectively, between the subsequent images. Image processing was performed using the Zeiss image analysis software (2.3.64.0). In brief, the multichannel images were first inspected with the bright field channel for focus, followed by the florescence count using the software’s “Image Analysis Wizard” using the GFP channel. The number of individual GFP regions represent the cell numbers, and the data were exported to Excel for the cell death calculation given in percentage. A step-by-step walk-through in the software can be found from the ZEISS’s ZEN 2 (blue edition) protocol (example 9.2, Counting number of fluorescence signals per nuclei).

### Microfluidic Phosphotriesterase Droplet Assay

#### Generation of First and Second Emulsion

Single cells were encapsulated into 7 pL droplets by mixing the cells suspension 1:1: on chip (three-inlet device) with an inducer-substrate solution (10 μM FddEP,^5,55^ mTA (final concentrations of 0, 5, 10, 15, and 125 μM), 50 μg mL^–1^ carbenicillin and 50 μg mL^–1^ kanamycin in LB) to a final OD_600_ of 0.208. As previously described,^7^ the aqueous phase from two aqueous flows was encapsulated into a fluorous oil phase (HFE-7500 (3M), 0.5% Fluoro-surfactant 008, (RAN biotechnology), 1.8 kHz) following a Poisson distribution with an expectation value λ of 0.29 (resulting in theoretically 75% of empty droplets, 22% with single cells, and 3% with multiple cells. The droplets were collected for around 40 min (approximately 4.5 million droplets) in a 1.5 mL Eppendorf tube on ice. For the second compartmentalization, the droplets were aspirated from the Eppendorf tube into oil-filled tubing and reinjected into a second microfluidic device to produce double-emulsions in an aqueous carrier phase (2% Tween 80, 100 mM Tris-HCl, pH 8.2) as water/oil/water compartments (see Figure S4) for approximately 30 min until all single emulsions aspirated into the tubing were re-emulsified. Double-emulsions for each inducer concentration were produced separately and collected on ice. The droplets were then incubated at 37 °C.

#### Measurement and Analysis

At each time point, 20 μL of emulsion were pipetted out and added to a well of a U-bottomed 96-well plate with 150 μL of buffer (1% Tween 80, 100 mM Tris, pH 8.2, 20% OptiPrep (Density Gradient Medium, Sigma-Aldrich)). The fluorescence of the double-emulsions was measured by flow cytometry (Guava EasyCyte, Merck-Milipore) and analyzed to obtain the graphs in Figure 6a (FlowJo, BD) and the percentage of droplets above the green fluorescence threshold of 200–800 RFU (Flowing Software, Opensource by Perttu Terho), determined at the minimum between the nonfluorescence droplet and fluorescent droplet populations, as these varied slightly for each condition (boxed area of Figure 6a i.e., 125 μM mTA has much higher background than 0 and 5 μM mTA).

### Cell Recovery Assay

The first emulsions were generated as described above and incubated (37 °C). At each time point, 20 μL of the emulsion was de-emulsified into recovery medium (20 μL of perfluoro-octanol [PFO], 100 μL of SOC [20 g L^–1^ tryptone; 5 g L^–1^ yeast extract; 2 mL of 5 M NaCl; 2.5 mL of 1 M KCl; 10 mL of 1 M MgCl_2_; 10 mL of 1 M MgSO_4_; 20 mL of 1 M glucose]). A 90 μL aliquot of this mixture was added to 810 μL of recovery medium for plating. This solution was used for further serial dilutions and spotting (10 μL, in triplicate) on kanamycin/carbenicillin agar plates (Figure S7). After spotting, the plates were left to air-dry (at room temperature, 10 min) before incubation (37 °C, overnight). The agar plates were imaged, and the colony forming units for each spot were counted and analyzed (Excel, MSOffice) to determine the average CFU for the from the readable 1:100, 1:1000, and 1:10 000 serial dilution spots.

## Supporting Information Available

Figures depicting measured LoD activities from single cells and in double emulsions, the overview of double emulsion setup, and videos showing the generation of double emulsions and detection of droplets. (PDF)
Videos showing the generation of double emulsions and detection of droplets (ZIP)

## Abbreviations

A.U.: arbitrary units
CV: coefficient of variation
CFU: colony forming unit
eDNA: environmental DNA
FddEP: fluorescein didiethyl phosphotriester
GFP: green florescent protein
IPA: isopropyl alcohol
LoD: lysis-on-demand
mTA: *meta*-toluic acid
OD: optical density
PFO: perfluoro-octanol
PTE: phosphotriesterase
PDMS: polydimethylsiloxane
PEI: polyethylenimine
SD: standard deviation.
