# Supplementary material for: A Titratable Cell Lysis-on-Demand System for Droplet-Compartmentalized Ultrahigh-Throughput Screening in Functional Metagenomics and Directed Evolution
Source: ACS Synth Biol. 2021 Jul 14;10(8):1882–94. doi: 10.1021/acssynbio.1c00084 (PMC8383311; doi:10.1021/acssynbio.1c00084)
Supplement: Supplementary file 1 — sb1c00084_si_001.pdf [file sb1c00084_si_001.pdf]

Supporting Information

A Titratable Cell Lysis-on-Demand System for Droplet-Compartmentalised Ultrahigh-Throughput Screening in Functional Metagenomics and Directed Evolution

Che Fai Alex Wong,[†] Liisa van Vliet,[‡] Swapnil Vilas Bhujbal,[†] Chengzhi Guo,[‡] Marit Sletmoen,[†] Bjørn Torger Stokke,[¶] Florian Hollfelder,[‡] and Rahmi Lale[*,†]

†*Department of Biotechnology, Faculty of Natural Sciences, Norwegian University of Science and Technology, Trondheim, N-7491, Norway*

‡*Department of Biochemistry, University of Cambridge, 80 Tennis Court Road, Cambridge, CB2 1GA, United Kingdom*

¶*Department of Physics, Faculty of Natural Sciences, Norwegian University of Science and Technology, N-7491, Trondheim*

E-mail: rahmi.lale@ntnu.no,RahmiLale

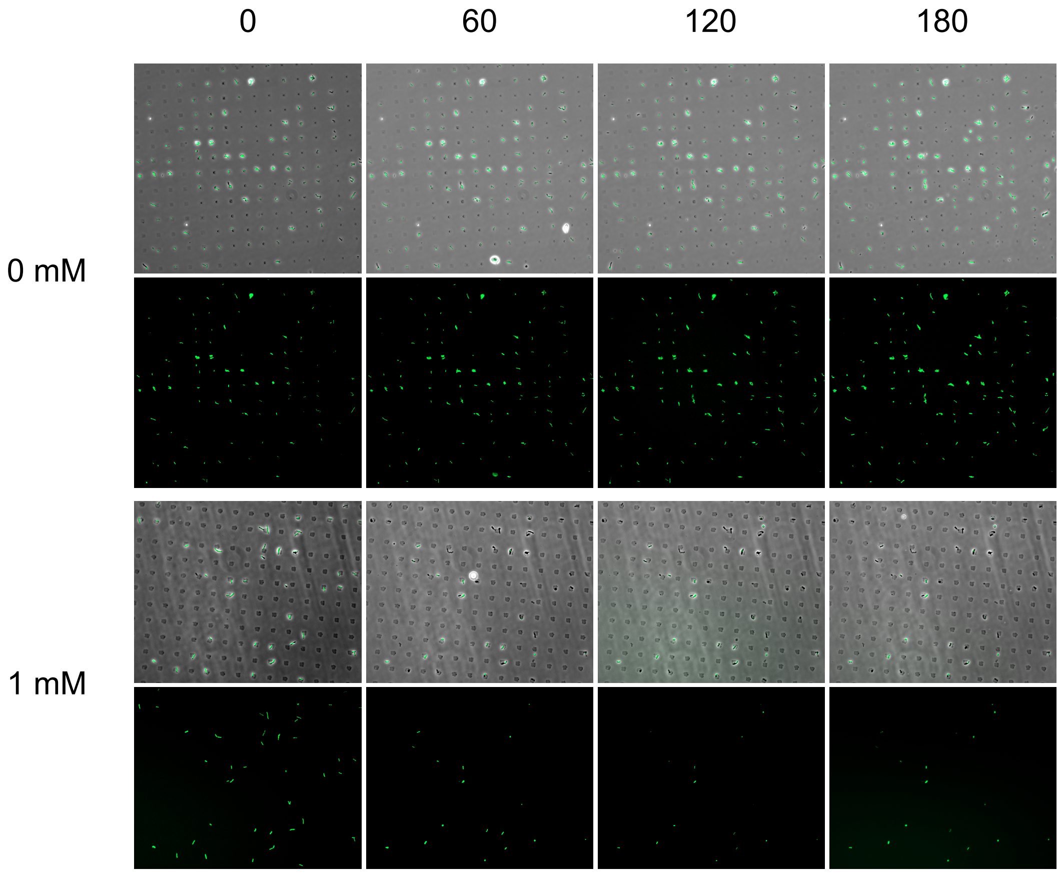

Figure S1: LoD activity measurement with immobilised single cells on µCP PEI-coated pattern. Time-lapse microscopic images for inducers concentrations 0 mM (row 1 and 2) and 1 mM (row 3 and 4) at every 60 minutes are depicted (row 1 and 3, overlaid bright-field and fluorescence images; row 2 and 4, fluorescence images). The number of *E. coli*-GFP-LoD cells, as counted from the GFP signals of the fluorescence images, remained ∼100% at 0 mM throughout the 180 minutes (row 2), whereas the cell number decreased to ∼40% at 1 mM induction from 60 minutes onward (row 4).

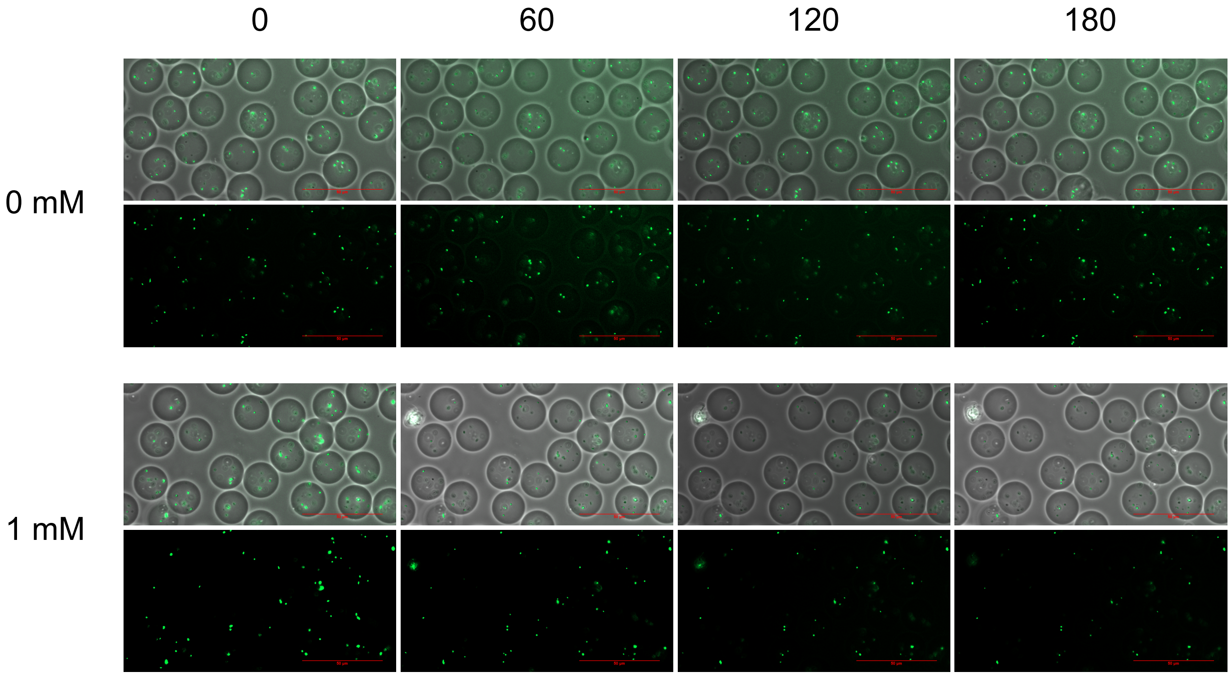

Figure S2: LoD activity measurement with encapsulated single cells in alginate microbeads. Time-lapse microscopic images for inducers concentrations 0 mM (row 1 and 2) and 1 mM (row 3 and 4) at every 60 minutes are depicted (row 1 and 3, overlaid bright-field and fluorescence images; row 2 and 4, fluorescence images). The number of *E. coli*-GFP-LoD cells, as counted from the GFP signals of the fluorescence images, remained ∼100% at 0 mM throughout the 180 minutes (row 2), whereas the cell number decreased to ∼50% at 1 mM induction by 180 minutes (row 4). Scale 50 µm.

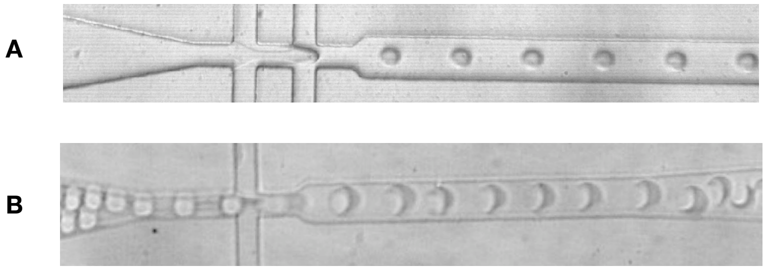

Figure S3: Generation of double emulsion. Stills of the supplemental videos SV1 and SV2. (A) the first water-in-oil emulsion generated 7 pL droplets in an oil carrier phase (1% surfactant in fluorinated oil) which are re-injected into (B) a hydrophilic flow-focusing device to generate double emulsions.

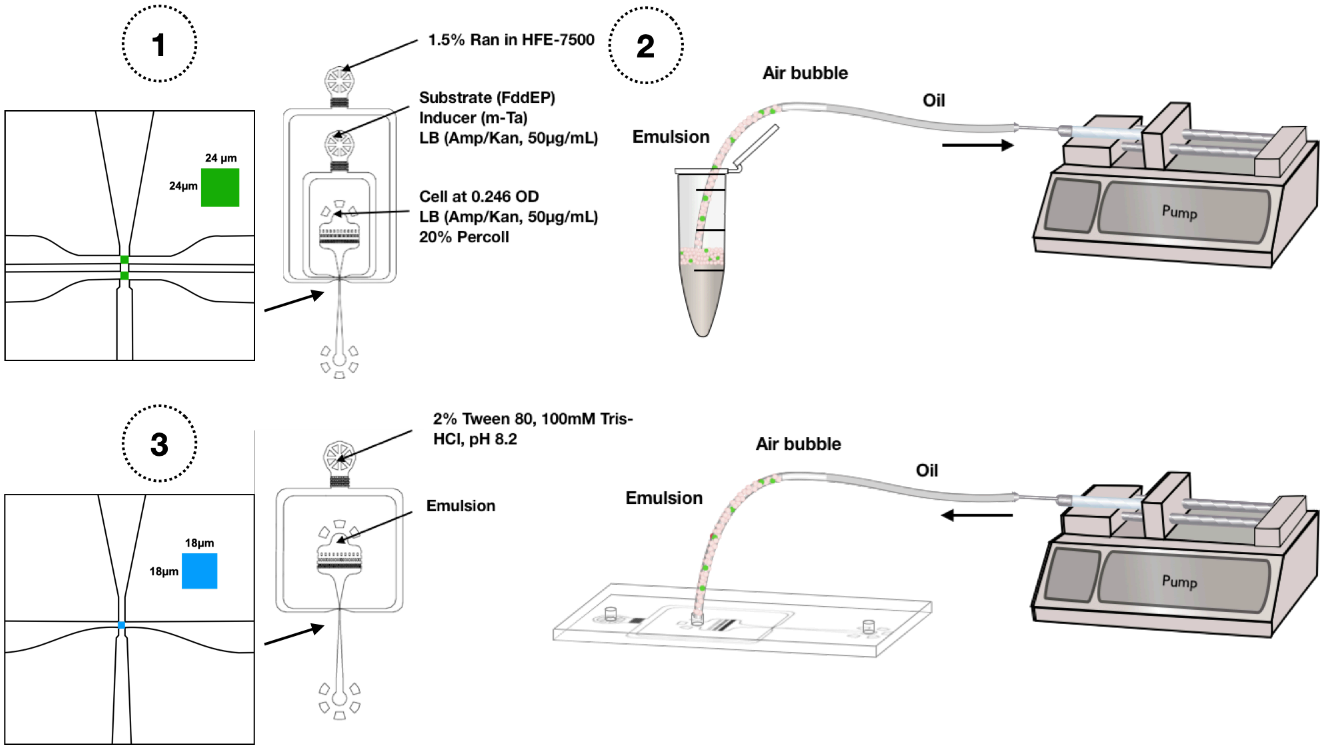

Figure S4: (1) The hydrophobic chip to generate the first emulsion in a triple inlet design with a flow-focusing junction of 24 µm. The carrier phase was a fluorous oil containing surfactant (1.5% surfactant in HFE-7500) (2) Collection of the emulsion by aspirating into an oil filed tubing and (3) Re-injection of the first emulsion into a two-inlet hydrophilic chip with a flow-focusing junction of 18 µm to generate double emulsion. The carrier phase is aqueous (2% Tween 80 in 100 mM Tris-HCl, pH 8.2).

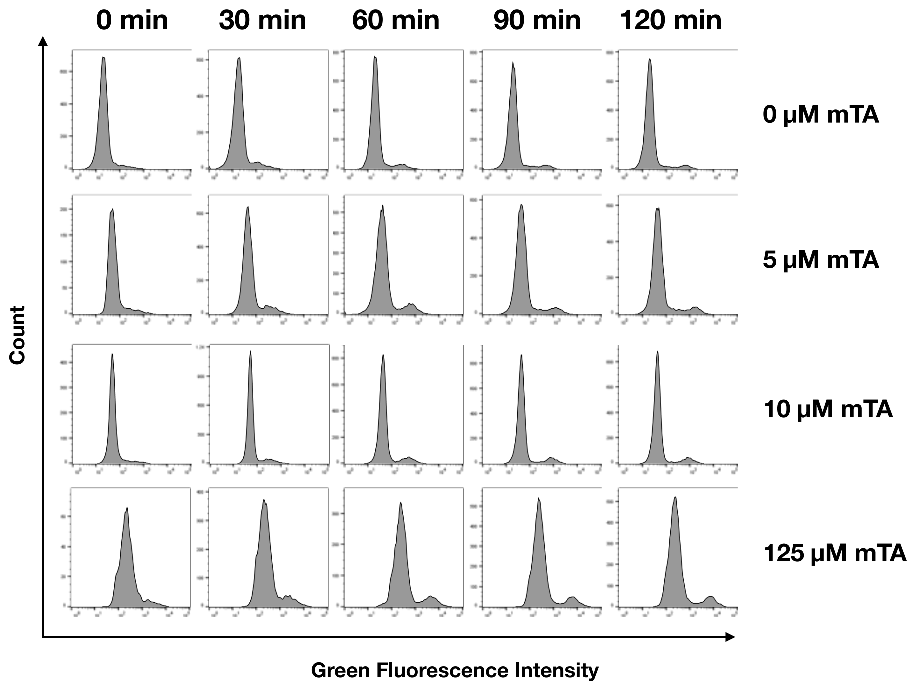

Figure S5: Individual flow cytometry time-course histograms for *E. coli*-PET-LoD cells incubated with 0, 5, 10 and 125 µM mTA. The PTE activity in the droplet population was measured at the start of the incubation at 37 °C every 30 minutes for 120 minutes. Each population represents approx. 10,000 events.

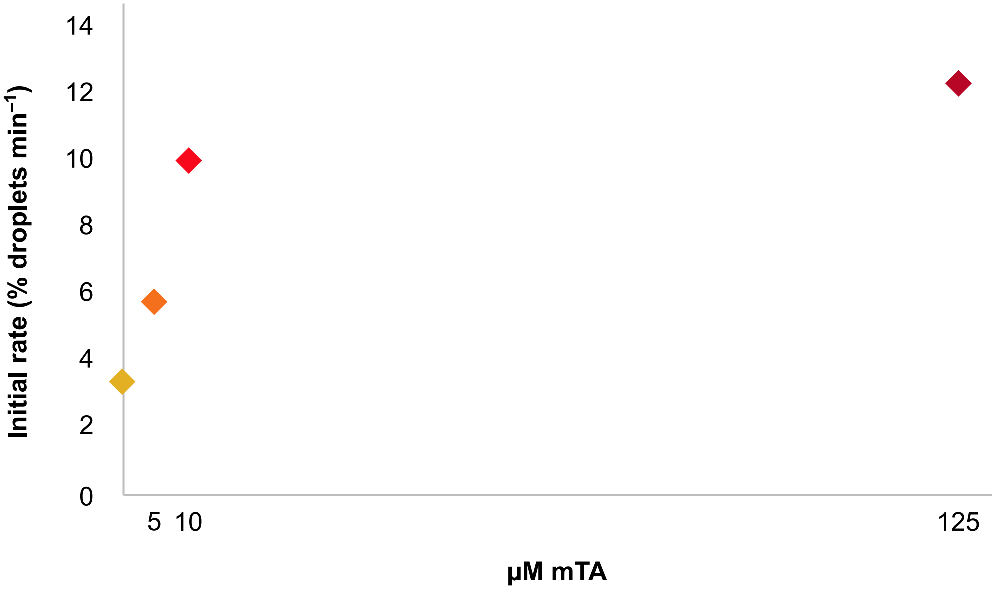

Figure S6: Initial rate of fluorescent droplets for *E. coli*-PET-LoD cells with varying mTA concentrations. The rate is expressed as the percentage of droplets turning over product per minute. The increase in the rate of droplets with fluorescent signal is dependant on mTA concentration suggests that the lysis of cells is titrateable.

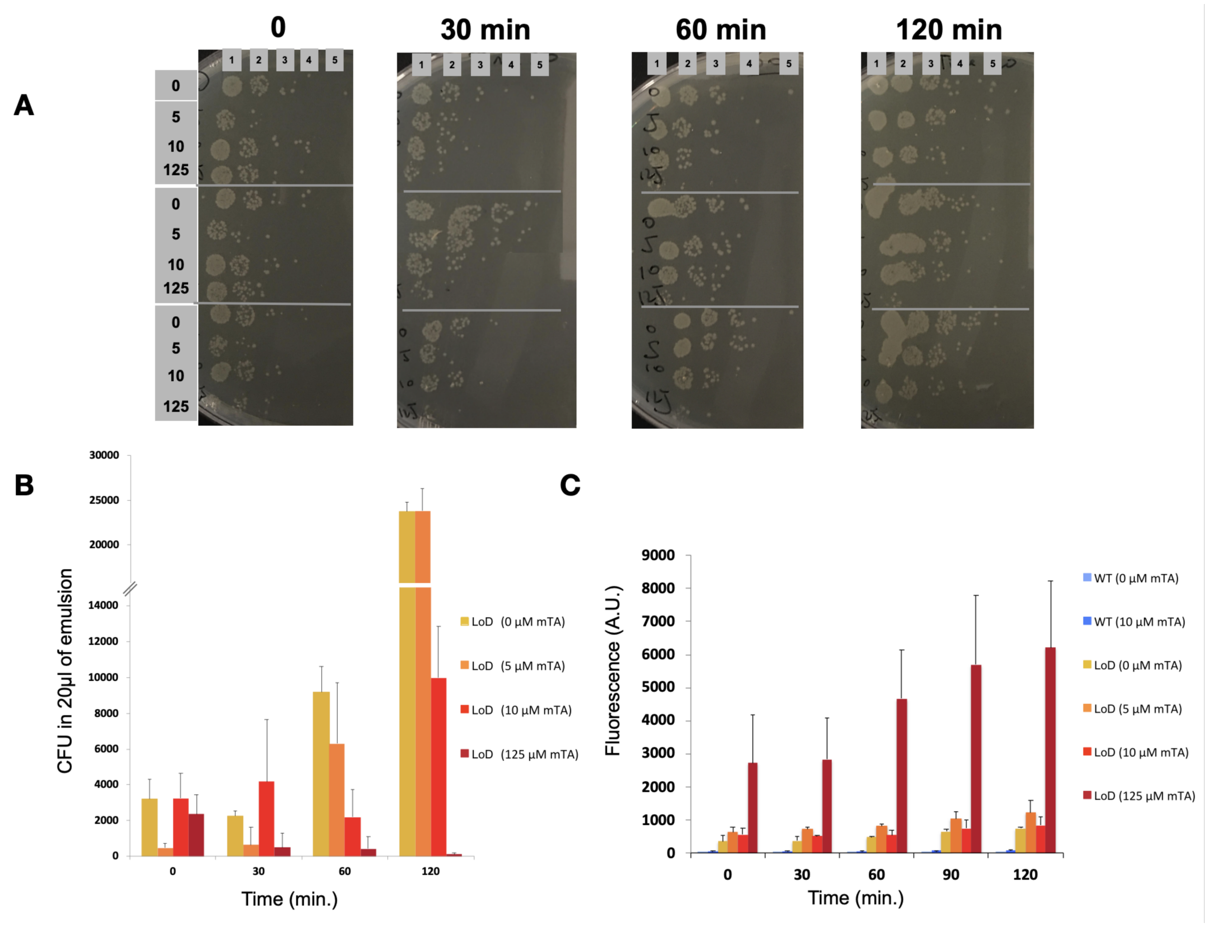

Figure S7: Recovery of cells from droplets. (A) Photographs of the agar plates used for cell growth with spot dilutions (in triplicate) for each mTA concentration and incubation time, grey lines separate the triplicate rows to guide the eye. The columns 1, 2, 3, 4, 5 represent 1:10, 1:10^2, 1:10^3, 1:10^4 and 1:10^5, respectively. (B) Number of colony forming units in 20 µL of emulsion (diluted 100 times) at different times of incubation (0, 30, 60, and 120 min), where the value represents the average CFU from all measurable dilution spots. (C) Fluorescence intensity of the droplets analysed in Figure 6 reflecting very high fluorescence or enzymatic turnover indicating complete lysis of cells for the WT controls (blue, 0 and 10 µM mTA) and the *E. coli*-PET-LoD cells (yellow-red for 0, 5, 10, and 125 µM mTA).

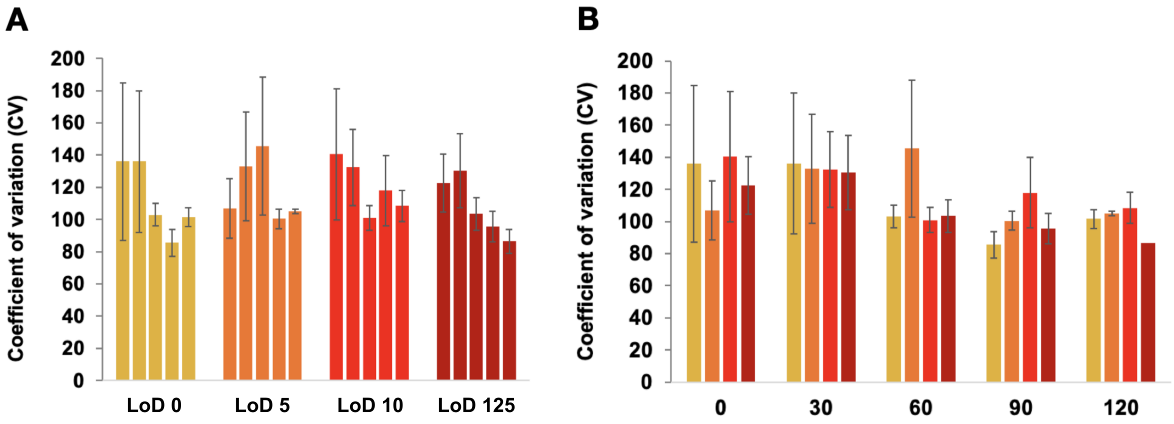

Figure S8: Coefficient of Variation (CV) for the high fluorescence populations in Figure 6. (A) Coefficient of variations for each inducer concentration, with sequential bars showing the time course (0, 30, 60, 90 and 120 min). The high-fluorescence populations coefficient does not vary much around a CV of 100, indicating that cell expression of PTE or cell growth is uniform across the droplets that contain lysed cells. Over time, the CV decreases slightly (last 3 columns of each condition), suggesting that expression levels of the LoD system is more uniform from about 60 minutes of incubation. The error bars represent the standard deviation from the 2 biological repeats each in triplicate that are described in Figure 6 (n=6). (B) The CVs are plotted over time with the colours representing the different [mTA] conditions, to help visualise the change in error bars: from 60 minutes, the CVs are slightly narrower, but also have less error between repeats (n=6), again suggesting that the distribution of LoD expression across the droplets is more uniform.

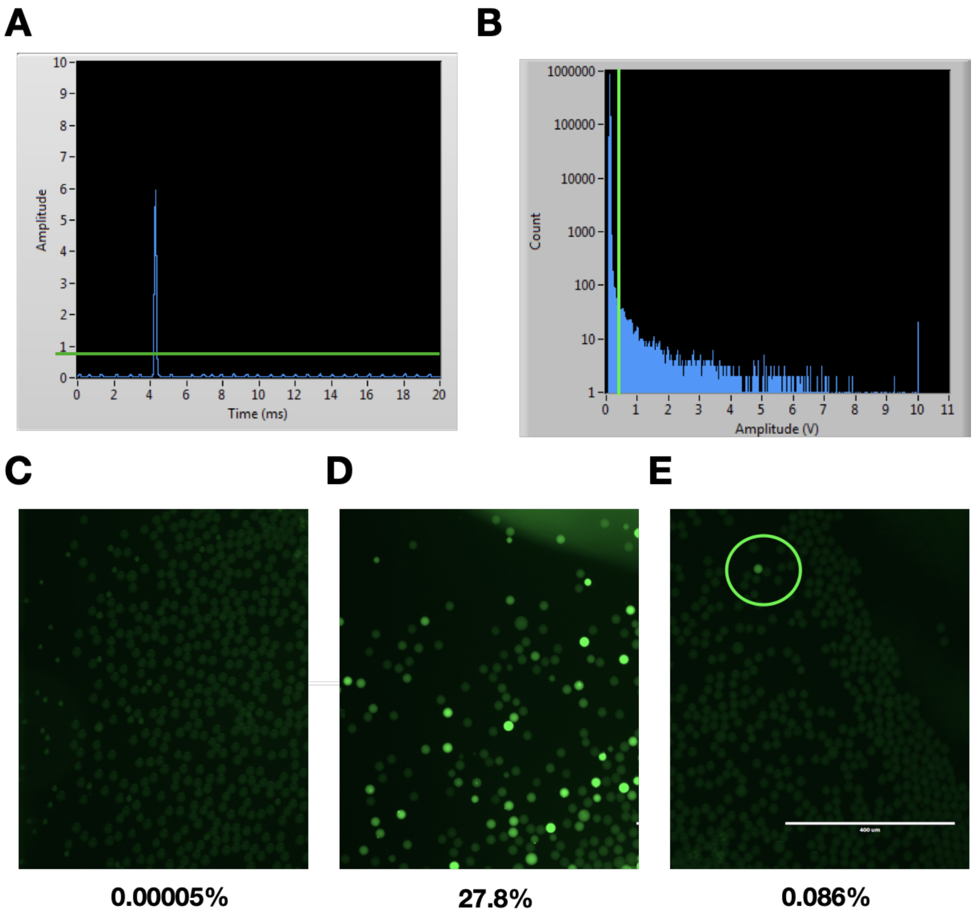

Figure S9: Detection of rare *E. coli*-PET-LoD cells within a population of WT cells. *E. coli*-PET-LoD cells were diluted 1:1000 with WT *E. coli*-PET cells and encapsulated at λ=0.3 (OD: 0.207) to simulate a library screening and detection in microfluidic FADS (Fluorescence Activated Droplet Sorting) chips as previously described.(*1–5*) (A) The fluorescence signal is represented by the amplitude (as voltage, V) of the signal detected. A 20 nM fluorescein off-set was added to the droplets to enable detection of each empty droplet (see video SV3), and the cells were induced with 125 µM of mTA, droplets were analysed at around 1kHz. Smaller peaks (each one a droplet) can be seen with one droplet containing high fluorescence. (B) A histogram of the droplet count (log scale) by fluorescence signal (or amplitude of the voltage signal) shows mostly empty droplets with a tail of fluorescence droplets. Overlaid bright-field and green fluorescence images of the WT (C), LoD only cells (D) and 1:1000 dilution (E) show that the LoD system can be used to detect and select droplets from a population containing rare active enzymes. The fluorescence signal threshold used to quantify the percentage of lysed droplets is shown with a green line in plots (A) and (B). Above this threshold (background of empty droplets, 0.00005% of WT *E.coli* cells were fluorescent, 27.8% of all *E. coli*-PET-LoD cells (or the majority of the theoretically 31% cell-containing droplets) and 0.086% or an estimated 1 in 340 cell-containing droplets. Nearly 6.3 million droplets (at 1:1,000 dilution) were analysed.

A

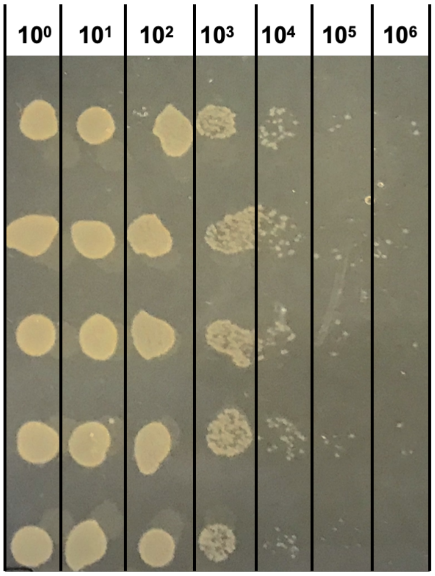

B

Dilution	10^0	10^1	10^2	10^3	10^4	10^5
1	-	-	-	-	**25**	3
2	-	-	-	-	**26**	4
3	-	-	-	-	**15**	3
4	-	-	-	-	**26**	5
5	-	-	-	-	**16**	6
**Count average**					**21.6**	4.2
St Dev. of count					5.6	1.3
**Count in 5µL**					**2.16E+05**	
**Count in 1mL**					**4.32E+07**	
**# 7.5pL droplets in 1mL**					**1.33E+08**	
**% droplet with cells**					**32.4%**	
St Dev. (% of droplets)					8.4%	

Figure S10: Determination of starting *E. coli*-PET-LoD cell concentration that was used in the 1:1,000 dilution assay (Figure S9). Freshly transformed *E. coli*-PET-LoD cells were diluted to OD: 0.207, for a theoretical droplet occupancy at λ=0.3 (or approximately cell in 3 droplets) based on an estimated 200 million BL21 cells for OD=1 5 µL of the cell solution (at the final cell concentration of droplets). (A) A photograph of the agar plate used for cell growth (CFU) with spot dilutions (5 replicates), with each column a 10-fold dilution from the previous, with 10^0 being the droplet concentration. (B) A table with the CFU counts that were used (from the 10^4 dilution were used) and calculations to estimate the empirical cell concentration of 43.2 10^8 cells/mL which would give a theoretical fill rate of 7.5 pL droplets (size of the droplets for the experiment in Figure S9 of 32.4% ± 8.4%. This correlates well with the theoretical fill of 31% (assuming OD=1 stocks contain 200 million cells), and 27.8% of droplets with fluorescence (for the LoD cells induced with 125 µM mTA) in Figure S9.

References

1. Colin, P.-Y., Zinchenko, A., and Hollfelder, F. (2015) Enzyme engineering in biomimetic compartments. *Current Opinion in Structural Biology 33*, 42–51.

2. Tauzin, A. S., Rangel Pereira, M., van Vliet, L. D., Colin, P.-Y., Laville, E., Esque, J., Laguerre, S., Henrissat, B., Terrapon, N., Lombard, V., Leclerc, M., Doré, J., Hollfelder, F. H., and Potocki-Veronese, G. (2020) Investigating host-microbiome interactions by droplet based microfluidics. *Microbiome 8*, 1–20.

3. Zurek, P. J., Knyphausen, P., Neufeld, K., Pushpanath, A., and Hollfelder, F. (2020) UMI-linked consensus sequencing enables phylogenetic analysis of directed evolution. *Nature Communications 11*, 1–10.

4. Zurek, P. J., Hours, R., Schell, U., Pushpanath, A., and Hollfelder, F. (2021) Growth amplification in ultrahigh-throughput microdroplet screening increases sensitivity of clonal enzyme assays and minimizes phenotypic variation. *Lab on a Chip 21*, 163–173.

5. Holstein, J. M., Gylstorff, C., and Hollfelder, F. (2021) Cell-free Directed Evolution of a Protease in Microdroplets at Ultrahigh Throughput. *ACS Synthetic Biology 10*, 252–257.
